# AllergoOncology: Emerging Translational and Clinical Significance of Basophils and Mast Cells in Cancer

**DOI:** 10.1002/clt2.70170

**Published:** 2026-04-16

**Authors:** Jack Alder, Katie E. Lacy, Debra H. Josephs, James Spicer, Heather J. Bax, Sophia N. Karagiannis, Jitesh Chauhan

**Affiliations:** ^1^ St. John's Institute of Dermatology School of Basic and Medical Biosciences & KHP Centre for Translational Medicine King's College London Guy's Hospital London UK; ^2^ School of Cancer and Pharmaceutical Sciences King's College London London UK; ^3^ Cancer Centre at Guy's Guy's and St. Thomas' NHS Foundation Trust London UK; ^4^ Breast Cancer Now Research Unit School of Cancer and Pharmaceutical Sciences King's College London Innovation Hub Guy's Cancer Centre London UK

**Keywords:** AllergoOncology, basophils, cancer immunotherapy, mast cells, tumor immunology

## Abstract

Mast cells and basophils, historically defined by their pathogenic roles in allergic diseases and type I hypersensitivity, are increasingly recognized as influential participants in cancer biology. Emerging research in AllergoOncology highlights their plasticity, diverse functions, and significance beyond classical contributions to allergy. This review summarizes current evidence on their presence, activation states, and roles across multiple cancer types. We examine their interactions with other immune populations, their context‐dependent pro‐ and anti‐tumor functions, and their potential utility as biomarkers. Their pro‐tumor activities include secretion of Th2 cytokines, release of angiogenic mediators, and facilitation of extracellular matrix remodeling, all of which can support tumor progression. Conversely, these cells may also promote anti‐tumor immunity through effector mechanisms and recruitment of cytotoxic CD8^+^ T cells. Translational tools such as the basophil activation test (BAT) and the mast cell activation test (MAT) are emerging to help predict hypersensitivities to cancer treatments including immunotherapies. A deeper understanding of their dynamic roles within the tumor microenvironment (TME) and across anatomical locations may reveal previously underappreciated functions, prognostic value, and therapeutic opportunities.

## 1 Introduction: Origin and Development of Human Basophils and Mast Cells

Two key populations are known to contribute to allergic responses, namely basophils and mast cells, whose roles in cancer and tumor immunity have increasingly come into focus within the emerging field of AllergoOncology. Constituting approximately 1% of all peripheral blood leukocytes in healthy individuals, basophils are the rarest granulocyte and are traditionally recognized for their critical involvement in hypersensitivity reactions and allergy responses via immunoglobulin E (IgE)‐ and non‐IgE‐mediated pathways [1]. They share phenotypic resemblance to other granulocytes, such as mast cells, which alongside their low frequency, has historically led to their underrepresentation in both experimental research and clinical contexts [2].

**FIGURE 1:**
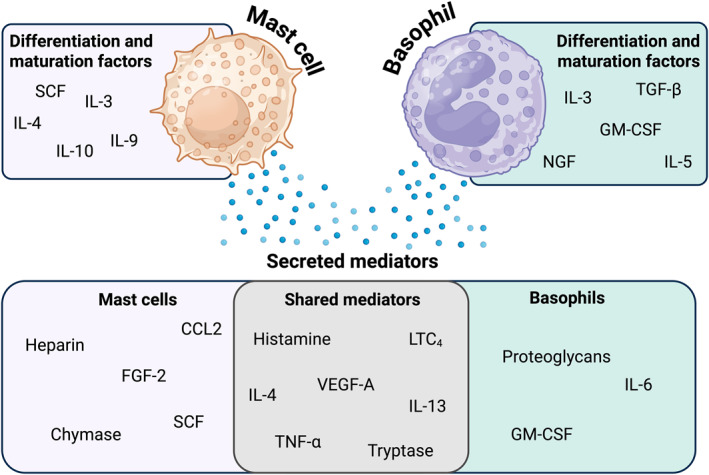
Mast cell and basophil heterogeneity in the production and secretion of priming factors and immune mediators. Schematic highlighting key differences and shared activation and effector functions between mast cells and basophils. Both cell types release mediators including histamine, leukotriene C_4_ (LTC_4_), vascular endothelial growth factor A (VEGF‐A), IL‐4, IL‐13, tumor necrosis factor‐α (TNF‐α), and tryptase. Mast cells additionally secrete heparin, CCL2, fibroblast growth factor 2 (FGF‐2), chymase, and stem cell factor (SCF). Whereas basophils additionally secrete proteoglycans, IL‐6, and granulocyte‐macrophage colony‐stimulating factor (GM‐CSF). Mast cell activation is primarily driven by SCF and a range of cytokines including interleukin (IL)‐3, IL‐4, IL‐9, and IL‐10. In contrast, basophils are responsive to a broader array of growth factors and chemokines that amplify their activation and mediator release. While both cell types can produce IL‐4 and IL‐13, this shared cytokine profile underscores shared and perhaps cooperative roles in type 2 inflammation despite distinct regulatory cues. Figure created in BioRender.

Mast cells are of myeloid lineage, reside within tissues, particularly at anatomical barrier sites such as the skin, intestines, and around blood vessels [3]. Mast cells, like basophils, are also involved in hypersensitivity and allergic reactions via IgE‐ and non‐IgE‐mediated mechanisms [4]. Mast cell progenitors arise from multipotent hemopoietic progenitor cells in the bone marrow. They enter the circulation as immature progenitors and subsequently home to peripheral tissues where they differentiate [5]. In tissues, mast cells differentiate and mature under local influences including stem cell factor (SCF) binding to the receptor c‐kit and cytokines such as interleukins (IL)‐3, IL‐4, IL‐9, and IL‐10 (Figure 1) [6, 7]. Notably, although SCF alone cannot trigger their degranulation, it significantly enhances mast cell activation and mediator production by facilitating IgE receptor (FcεRI) crosslinking [8].

Basophils differentiate in the bone marrow. IL‐3 has been identified as the most potent differentiation factor for human basophils, through binding to its highly expressed cell surface receptor CD123 (IL‐3R) [9]. Other cytokines and growth factors, such as IL‐5, granulocyte‐macrophage colony‐stimulating factor (GM‐CSF), transforming growth factor‐beta (TGF‐β), and nerve growth factor (NGF), have also been shown to support basophil differentiation and development (Figure 1) [9]. TGF‐β is reported to promote IL‐3‐induced basophil differentiation [10], whereas IL‐5 and NGF can act synergistically with GM‐CSF to augment basophil differentiation from progenitor myeloid cells [11]. Upon activation, basophils release histamines, proteoglycans, pro‐inflammatory mediators (vascular endothelial growth factor A [VEGF‐A], leukotriene C4 [LTC_4_]), and T helper 2 (Th2)‐type and immunoregulatory cytokines, such as IL‐4, IL‐6, IL‐13, GM‐CSF, and tumor necrosis factor‐α (TNF‐⍺) (Figure 1) [12, 13].

Mast cells are tissue‐resident cells, known for their contributions to inflammatory cascades [7, 8]. Activation via both innate or adaptive immune mechanisms prompts mast cell degranulation through secretory granules containing preformed inflammatory mediators, including cytokines, and eicosanoids, as well as pro‐angiogenic factors such as fibroblast growth factor‐2 (FGF‐2) [14]. Mast cell activation and degranulation can be assessed and monitored through histamine and tryptase release [15] (Figure 1).

**FIGURE 2:**
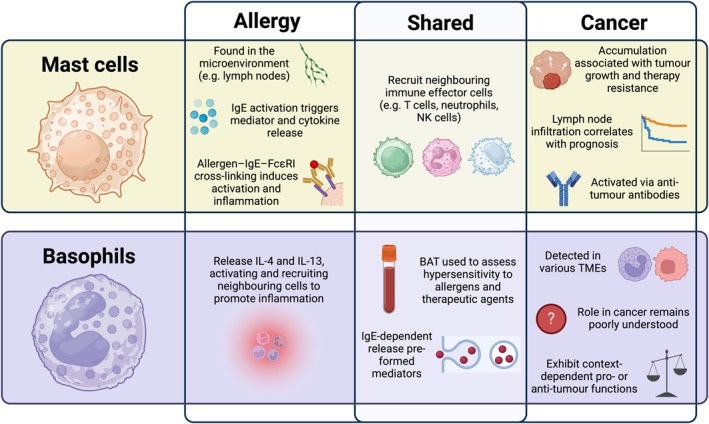
Basophils and mast cells and their comparative traits and functions in allergy and in cancer. Both cell types display features but overlapping roles in allergy and cancer‐related processes in the field of AllergoOncology. BAT, basophils activation test; TME, tumor microenvironment.

While basophil and mast cell activation and degranulation are well‐established contributors of allergic disease, this review focuses on their activation states and functions in cancer. Basophils and mast cells are central to AllergoOncology, not just due to their classical roles as immune effector cells in IgE‐mediated immune responses, but also due to their unique immunomodulatory capabilities that allow them to actively shape peripheral immune responses and inflammation and impact the tumor microenvironment (TME). We highlight multiple roles of these granulocytes, including influencing pro‐tumor systemic and local immune environments through their secretion of Th2 cytokines, release of angiogenic mediations, and facilitating extracellular matrix remodeling (ECM), that support tumor progression. We furthermore discuss their juxtaposed roles in promoting anti‐tumor immunity via pro‐inflammatory and effector mechanisms and through recruitment of cytotoxic CD8^+^ T cells. Within the context of AllergoOncology, which explores the immunological interface between allergy, IgE‐mediated immune responses, and oncology, we highlight translational opportunities and identify key areas for future investigation in cancer prognosis and therapy (Figure 2).

## 2 Basophil and Mast Cell Activation Tests Applied in Allergic Disease and Translated to Cancer

*Basophils:* Basophils in human circulation are involved in allergic reactions such as in hypersensitivities and anaphylaxis [13, 16]. Like other granulocytes, they migrate from the blood compartment to inflamed tissues acting as allergic cells [13]. They can secrete VEGF, a potent inducer of endothelial cell proliferation and migration to sites of inflammation [17].

The basophil activation test (BAT) is becoming an established tool in the study of basophils and widely used to predict type I hypersensitivity reactions to food, venom, and drugs within the field of allergy, and beyond [18, 19, 20, 21, 22]. This flow cytometric assay measures basophil activation, assessed as the upregulation of cell‐surface activation markers, most commonly CD63 and CD203c, following ex vivo incubation of whole unfractionated blood with a potential allergen to assess basophil activation propensity, and thus potential of a type I hypersensitivity reaction to a given agent [18, 23]. The application of the BAT within the field of AllergoOncology is emerging; it has been demonstrated to help predict and monitor hypersensitivity responses to anti‐cancer drugs, including chemotherapeutics and monoclonal antibodies such as the anti‐EGFR antibody cetuximab [24, 25].

The role of basophils in the circulation of cancer patients and their propensity to be stimulated ex vivo has been evaluated [26]. The propensity of basophils from cancer patients for activation was studied ex vivo by measuring CD63 cell surface expression using the BAT. Irrespective of prior anti‐cancer treatment, including chemotherapy, targeted treatments, or biologics, basophils could be activated by IgE‐ and non‐IgE‐mediated triggers [27]. This suggests that the activation capacity of circulating basophils in cancer patients is preserved despite exposure to systemic therapies.

Several studies have also shown that it is possible to study basophils ex vivo to confirm, monitor, or predict hypersensitivity reactions in patients in response to novel therapeutic agents. With regards to the emergence of anti‐cancer IgE class therapeutics, there is a perceived risk of induction of adverse allergic reactions such as type I hypersensitivity and resultant anaphylaxis, when treating patients with IgE‐based immunotherapy [28]. Therefore, the BAT has been applied to evaluate patients for the potential of a hypersensitivity reaction to this novel immunotherapy class prior to their treatment [28, 29]. The BAT has demonstrated that IgE‐based therapeutics can be administered to most patients with cancer, with likely fewer than 5% being potentially hypersensitive to anti‐cancer IgEs [28, 29].

*Mast cells:* Mast cells are predominantly found within tissues, where they are responsible for immediate allergic reactions [30]. They cause allergic symptoms through degranulation and subsequent release of mediators, including histamine, cytokines (e.g., SCF, IL‐3, IL‐4, IL‐9, and IL‐10), and inflammatory mediators such as heparin and chondroitin sulfate [31, 32]. Activation and degranulation can occur when an allergen crosslinks allergen‐specific IgE bound to high‐affinity IgE receptors, FcεRI, expressed on the mast cell surface [33]. IgE‐mediated mast cell responses can mobilize dendritic cells to lymph nodes and promote Th2 polarization through the release of IL‐4 and IL‐13 [34]. Similarly to the BAT, the mast cell activation test (MAT) is a novel experimental flow cytometric assay for exploring mast cell reactivity, currently used to assess responses to allergens [35]. Although less developed than the BAT, the MAT may find applications to cancer in future and could be applied to better understand mast cells, their activation states, and functional trigger mechanisms within a cancer setting.

*Key barriers for implementation of basophil and mast cell tests in AllergoOncology:* Despite their translational promise, several barriers currently limit the routine clinical implementation of the BAT and MAT [18, 22, 36, 37]. These include lack of harmonized protocols, variability in activation markers (e.g., CD63 vs. CD203c), absence of universally accepted cut‐off values for detecting activation, inter‐laboratory variability, requirement for fresh sample for functional evaluation, and recognized issues with a proportion of individuals who fall into “non‐responder” or “non‐releaser” categories for whom these tests are not informative. For MAT, additional heterogeneity arises from differences in the different sources of mast cell sources and variable assay platforms [38]. Broader clinical integration of these tools will require analytical and clinical validation, quality assurance frameworks, and consensus‐based standardization efforts, such as those underway within the AllergoOncology EAACI Working Group's Task Forces [22]. Realistically, implementation may initially be achieved through collaboration between specialized oncology centers and experienced flow cytometry laboratories and facilities, followed by phased integration into prospective clinical trials once reproducibility and regulatory standards are established. Notably, the BAT has already been incorporated into early‐phase clinical evaluation of therapeutic anti‐cancer IgE antibodies, including the completed and the ongoing clinical trials of MOv18 IgE in patients with solid tumors, to prospectively assess potential hypersensitivity risk and to support translational safety monitoring [23, 39].

## 3 Basophil and Mast Cell Infiltrates in the Tumor Microenvironment (TME)

*Basophils:* Limited research has focused on basophil infiltration and function in solid tumors, including melanoma, colorectal, pancreatic, lung and gastric cancers (summarized in Table 1) [27, 49, 66, 67]. Basophil recruitment into tumors has been reported in animal models, and basophil and activated basophil signatures are associated with differential prognosis among various human cancers [27, 44]. However, the precise mechanisms by which basophils interact within the TME have yet to be fully uncovered. In murine models of melanoma, regulatory T cell (Treg) depletion was associated with increased basophil and CD8^+^ T cell infiltration, and basophils within the TME may potentially exert varying pro‐tumor signals [44]. Basophil infiltration has also been identified in human non‐small cell lung cancer, accounting for 0.4% of tumor leukocytes, as identified by flow cytometry [68].

**TABLE 1:** Context‐dependent roles of mast cells and basophils across tumor types.

Cancer type	Cell type	Pro‐tumor evidence	Anti‐tumor evidence	Predomidant functional tendency	Clinical implications
Melanoma/skin	Mast cells	IL‐33/ST2 receptor signaling implicated in progression; C3‐high mast cell phenotype associated with poor survival; humanized mouse models show reduced CD8^+^ infiltration and resistance to anti‐PD‐1; micro‐regional clustering linked to immune exclusion [40, 41]	Complement activation may mediate tumor cytotoxicity (CDC); context‐dependent anti‐tumor activity reported [42, 43]	Predominantly pro‐tumor in advanced disease	Associated with resistance to immune checkpoint blockade; preclinical data suggest c‐Kit inhibition may restore anti‐PD‐1 responsiveness [41]
	Basophils	Limited evidence	Treg depletion associated with basophil and CD8^+^ infiltration; CCL3/CCL4‐secreting basophils promote CD8^+^ recruitment; IL‐33 stimulation induces granzyme B [4]	Predominantly anti‐tumor (preclinical)	Potential enhancer of cytotoxic T‐cell responses; clinical validation required
Pancreatic (PDAC)	Mast cells	Promote tumor growth and invasion; mast cell‐conditioned media induces proliferation via MMP activity; pro‐angiogenic mediator release (VEGF, FGF‐2, tryptase) [45, 46]	Limited evidence	Predominantly pro‐tumor	Mast cell depletion improves survival in models; high infiltration associated with poorer outcomes [47, 48]
	Basophils	Recruited to TDLNs via CCL7/MCP3; IL‐3 activation leads to IL‐4 release and Th2‐skewed microenvironment [49]	Limited evidence	Predominantly pro‐tumor	High basophil proportion in TDLNs associated with poorer post‐surgical survival [49]
Lung (LUAD/NSCLC)	Mast cells	Lung cancer–derived exosomes activate mast cells; SCF–KIT signaling induces tryptase release and pro‐angiogenic remodeling; pro‐coagulant mediator release; TAMC heterogeneity (CD103^+^/CD103^−^ subsets) described [50, 51, 52]	Higher total mast cell density associated with improved OS and PFS in early‐stage LUAD [53]	Mast cell abundance and phenotype correlate with prognosis; potential biomarker relevance	Mast cell abundance and phenotype correlate with prognosis; potential biomarker relevance
	Basophils	Galectin‐3–mediated activation induces IL‐4/IL‐13 release; may support Th2‐polarized inflammation [54]	High expression of CD123, CCR3, FcεRI associated with improved OS [44]	Likely anti‐tumor (associative clinical data)	Basophil marker expression linked to improved survival
Gastric	Mast cells	Promote angiogenesis (VEGF, FGF‐2, CXCL8); activate TNF‐α and PD‐L1 pathways; tumor‐derived adrenomedullin induces degranulation; protease‐mediated matrix remodeling [55, 56]	Tryptase density associated with improved OS/PFS in early‐stage disease [57]	Predominantly pro‐tumor (stage‐dependent)	Associated with tumor progression and immune suppression; PD‐L1 modulation suggests therapeutic relevance
	Basophils	Peripheral counts correlate with tumor infiltration; proximity to M2‐like macrophages; associated with Th2‐polarized environment [58]	Limited evidence	Likely pro‐tumor (associative)	Peripheral basophil levels may reflect tumor immune status
Colorectal	Mast cells	Involved in angiogenesis, lymphangiogenesis, and interactions with macrophages, T cells and MDSCs [59, 60]	Some studies report favorable prognostic associations	Heterogeneous	Prognostic significance remains controversial
	Basophils	Involved in angiogenesis, lymphangiogenesis, and interactions with macrophages, T cells and MDSCs [59, 60]	Higher circulating basophils associated with improved outcomes	Predominantly anti‐tumor (clinical correlation)	Basopenia associated with poor prognosis [61]
Ovarian	Mast cells	Limited evidence	Mast cell recruitment into TME associated with favorable outcomes	Predominantly anti‐tumor	Correlates with improved survival
	Basophils	Limited evidence	Higher circulating basophils; preserved ex vivo BAT responsiveness (CD63 upregulation); tumor expression of CCR3, CD123, FcεRI, CD63, CD203c, tryptase; activated transcriptomic signatures associated with improved survival [27]	Predominantly anti‐tumor	Elevated basophil numbers and activation associated with favorable prognosis
Breast	Mast cells	Some studies associate high mast cell density with poor prognosis [62]	Other studies show favorable prognostic roles, particularly stromal localization [63]	Context‐dependent	Prognostic role remains debated
	Basophils	Insufficent data	Insufficient data	Not established	Clinical relevance unclear
Esophageal adenocarcinoma	Mast cells	Limited evidence	Recruitment correlated with favorable outcomes [64]	Predominantly anti‐tumor	Associated with improved prognosis
	Basophils	Insufficient data	Insufficient data	Not established	Clinical relevance unclear
Diffuse large B cell lymphoma (DLBCL)	Mast cells	Limited evidence	Recruitment associated with favorable outcomes [65]	Predominantly anti‐tumor	Associated with improved survival
	Basophils	Insufficient data	Insuffient data	Not established	Clinical relevance unclear

*Note:* Evidence type note: mechanistic findings are derived from a mixture of human datasets (e.g., transcriptomics, CyTOF, clinical cohorts) and preclinical models (murine and in vitro). Where evidence is predominantly preclinical or associative, this is indicated in the “predominant functional tendency” and/or “clinical implications” columns.

Abbreviations: BAT, basophil activation test; CCL, C‐C motif chemokine ligand; CDC, complement‐dependent cytotoxicity; DLBCL, diffuse large B‐cell lymphoma; FGF‐2, fibroblast growth factor 2; KIT, KIT receptor; LUAD, lung adenocarcinoma; MCP‐3, monocyte chemotactic protein‐3; MDSCs, myeloid‐derived suppressor cells; MMP, matrix metalloproteinase; NSCLC, non‐small cell lung cancer; OS, overall survival; PD‐1, programmed cell death protein‐1; PDAC, pancreatic ductal adenocarcinoma; PD‐L1, programmed death ligand‐1; PFS, progression‐free survival; SCF, stem cell factor; TAMCs, tumor‐associated mast cells; TDLNs, tumor‐draining lymph nodes; TME, tumor microenvironment; TNF‐α, tumor necrosis factor alpha; Tregs, regulatory T cells.

*Mast cells:* Tumor‐infiltrating mast cells have been identified within the TME of numerous cancer types including melanoma, colorectal, pancreatic, lung, and gastric cancers, and are gaining attention for diverse roles in cancer progression and prognosis [50, 69, 70, 71, 72]. They may be recruited into the TME through various growth factors and chemokines including SCF, VEGF, and chemokine (C‐C motif) ligand 2 (CCL2) [73, 74] and are reported to display both pro‐ and anti‐tumor behaviors via differing immunomodulatory effects upon activation and degranulation. In general, there is a large variability in mast cell phenotype and function, and the extent by which they can produce and release mediators such as histamine, inflammatory mediators, and cytokines. This may depend on their anatomic location, exposure to local inflammatory mediators, and stage of mast cell development. Specifically, mast cell recruitment in the TME has been correlated with positive outcomes in some cancers, such as ovarian cancer, diffuse large B cell lymphoma, and esophageal adenocarcinoma [63, 64, 65], but conversely, with poor prognosis in other cancer types such as human melanoma, gastric, breast, and lung cancers [75, 76, 77, 78]. In breast cancer, the prognostic significance of mast cells remains controversial; some studies associate high mast cell density with poor prognosis, whereas others report favorable prognostic roles, particularly with mast cell infiltration within the tumor stroma [62, 79]. In immune checkpoint blockade (ICB) treatment, higher infiltration of mast cells was reported as a predictor of poorer response to anti‐programmed cell death‐1 (PD‐1) ICB in patients with melanoma [78].

**FIGURE 3:**
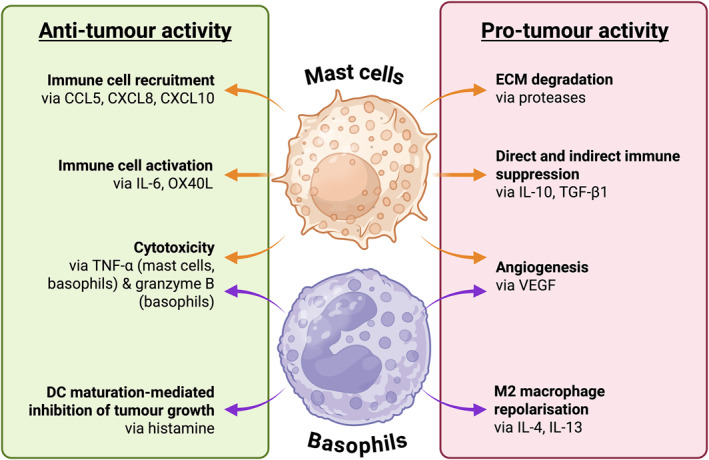
Dual roles of mast cells and basophils in the tumor microenvironment (TME). Schematic summarizing the context‐dependent pro‐ and anti‐tumor functions of mast cells and basophils mediated by distinct cytokines, chemokines, and effector molecules. Left: anti‐tumor functions include cytotoxicity (often mediated by soluble pro‐inflammatory mediators, e.g. TNF‐α, granzyme B), recruitment and activation of immune cells through chemokine gradients (e.g., CCL5, CXCL8, CXCL10, IL‐6, OX40L), and dendritic cell maturation associated with inhibition of tumor growth (e.g., histamine). Right: pro‐tumor activities include promotion of angiogenesis (via secretion of e.g. VEGF), extracellular matrix degradation (e.g., by protease release), immune suppression (e.g., IL‐10, TGF‐β1), and induction of M2‐like alternatively activated macrophage polarization by basophil‐derived IL‐4 and IL‐13. The balance between these juxtaposing activities is influenced by local microenvironmental cues and may determine disease progression and therapeutic response across different disease settings. Figure created in BioRender.

Together, these findings point to heterogeneous roles of basophils and mast cells within the TME, where their presence may contribute to either pro‐ or anti‐tumor immune modulation depending on the cancer type and microenvironmental context (Figure 3). The complexity of their functions and interaction remains incompletely understood, warranting further in‐depth investigation.

## 4 Differences Between Tissue‐Resident and Peripheral Blood Basophils

Basophils have traditionally been viewed as circulating cells that are rarely present in tissues; however, this concept is now being challenged. For example, in mice, basophil infiltration was detected throughout all stages of lung development [80]. Lung‐resident basophils are localized within the alveoli and exhibit a phenotype distinct from that of peripheral blood basophils. They are reported to be essential for both the transcriptional and functional development of alveolar macrophages, driving their polarization towards an anti‐inflammatory M2‐like state. These findings raise the possibility that basophils contribute to regulation of tumor‐associated macrophage activity. It has been proposed that tissue‐resident basophil gene expression profiles can be modulated within the TME by cytokines, such as IL‐33 and GM‐CSF [80]. Collectively, these studies point to diverse roles of circulating versus tissue‐resident basophils as active regulators of tumor outcomes through pro‐ or anti‐tumor functions.

## 5 Roles of Basophils and Mast Cells in the TME

*Basophils:* Basophils within the TME, studied through transcriptomic analyses of human tumors and in animal models, have been shown to exert both tumor‐promoting and tumor‐suppressing properties, depending heavily on the cancer type and local context (Figure 3).

Evidence from murine models suggests that basophils can support tumor progression through the secretion of Th2‐associated mediators, including IL‐4, IL‐6, IL‐13, CD40L, VEGF‐A/B, B cell activating factor, and histamine. Among these, IL‐4 and IL‐13, long recognized as hallmarks of allergic diseases, are also thought to drive the pro‐tumor activities of basophils [55, 81, 82]. Cytokines such as IL‐33, which are upregulated during chronic inflammation and may also be abundant within the TME, can activate basophils to release IL‐4 and Th2‐related mediators. In the human lung, basophil‐derived IL‐4 has been shown to support alternatively activated M2‐like macrophage phenotypes characterized by *Arg1, Clec7a,* and *Itgax* expression [80, 83]. It is plausible that a similar axis could contribute to the tumor‐promoting immunological landscape, although this remains to be directly demonstrated.

Conversely, basophils are also capable of exerting anti‐tumor functions through the release of inflammatory mediators, such as TNF‐⍺ and tryptase [84, 85, 86, 87]. These roles have been underscored in basophil‐deficient mouse models, particularly in melanoma, where their absence is associated with accelerated tumor progression [44, 67]. In contrast, findings from experimental and clinical studies in a pancreatic mouse model of cancer indicate a predominantly pro‐tumor role for basophils. Together, these observations highlight the importance of the tumor‐specific context in determining whether basophils act to promote or suppress cancer [49, 67]. Their duality may partly reflect the interplay between mediators with opposing functions: pro‐inflammatory, anti‐tumor factors such as TNF‐⍺ and pro‐tumor, pro‐angiogenic factors such as VEGF [88, 89, 90].

*Mast cells:* Mast cells may influence tumor progression by promoting angiogenesis and lymphangiogenesis (Table 1) [45, 91]. They may also actively modulate the TME through interactions with neighboring immune cells, such as B cells and T cells, via soluble mediators and direct receptor‐ligand contact, such as CD40L‐CD40 and OX40L‐OX40 pathways, while cytokines such as IL‐4, IL‐6, and TNF‐⍺ regulate recruitment, development, and survival [92]. Mast cells produce several cytokines including IL‐4, IL‐5, IL‐6, and IL‐13, which influence B cell differentiation [93]. Crosstalk between mast cells and B cells via the CD40L‐CD40 axis can induce class switching to IgE in the presence of IL‐4, even in the absence of T cell help [93]. Co‐culture of Tregs with murine bone marrow‐derived mast cells suppresses degranulation, but primes mast cells for IL‐6 production, which has been demonstrated to play a critical role in the expansion and differentiation of tumor cells [94].

Notably, mast cells, and to some extent basophils, display context‐dependent duality, with pro‐ and anti‐tumor activities influenced by cancer type, disease stage, and local microenvironmental signals (Figure 3).

The pro‐tumor activity of mast cells is supported by correlations between mast cell infiltration and disease progression across several solid tumors [95]. Mast cells facilitate angiogenesis through the secretion of VEGF, thereby enhancing endothelial cell proliferation and tumor vascularization. They also contribute to tumor invasion by releasing proteases, such as chymase and tryptase, which degrade ECM components, including collagen and fibronectin [96], while simultaneously mobilizing tumor promoting growth factors, such as VEGF and TGF‐β1. Additionally, mast cells support immune evasion through the release of immunosuppressive IL‐10 [81]. Histamine represents the most well‐described mast cell mediator. Activation of the H4 histamine receptor has been shown to drive epithelial‐mesenchymal transition (EMT) and ECM degradation, whereas pharmacological inhibition of histamine synthesis suppresses tumor growth [97]. In melanoma‐bearing mice, antagonism of HRH1 receptor suppressed mast cell infiltration, decreased VEGF levels and inhibited tumor progression by downregulating hypoxia‐inducible factor‐1 alpha (HIF‐1⍺) [98]. Similarly, in mouse models, blockade of the HRH2 receptor was associated with reduced tumor growth [99].

Beyond these preclinical findings, histamine receptors (H1‐H4) are expressed in tumors most likely by cancer cells and are thought to regulate cell proliferation, invasion, vascularization, and apoptosis [100, 101]. Furthermore, elevated histamine levels have been reported in various human cancers, including melanoma, colon, and breast tumors [102]. Mechanistically, histamine binding to HRH1 receptors within the TME suppresses CD8^+^ T cell activity, thereby promoting tumor growth and treatment resistance. Inhibition of this pathway with HRH1 antagonists can restore CD8^+^ T cell function and enhance immunotherapeutic efficacy [103]. In a retrospective cohort study of patients with stage IV lung cancer receiving H1 antihistamines alongside immune checkpoint inhibitors (ICIs) demonstrated significantly improved overall survival (OS) and progression‐free survival (PFS) compared to patients receiving ICIs, highlighting the potential of antihistamine to augment ICI therapy [104].

Mast cells also exhibit anti‐tumor activity. They can directly induce tumor cell death through the release of TNF‐⍺ or act indirectly via heparin secretion which modulates fibroblast function and interferes with angiogenesis [92, 105]. A key mechanism involves the inhibition of fibroblast growth factor (FGF) signaling. FGFs, together with VEGF, are major regulators of tumor angiogenesis and progression, acting through multiple pathways including PI3K‐AKT, PLC, and RAS‐MAPK cascades [106, 107]. Heparin binds and sequesters FGFs, preventing receptor engagement, thereby attenuating downstream signaling and limiting tumor vascularization and survival [107, 108].

Taken together, these findings reinforce the multifaceted and context‐dependent nature of mast cell biology within the TME. Their capacity to either enhance or restrain tumor development underscores the need for precise understanding of their signaling pathways and receptor interactions when evaluating prognostic and therapeutic relevance.

To illustrate this complexity, the following sections examine distinct cancer types in which mast cells and basophils have been reported to exert divergent or context‐specific roles. Skin, pancreatic, lung, gastric and colorectal cancers were selected as representative examples since they encompass both protective and tumor‐promoting activities, highlight interactions with key immune and stromal components and are supported by substantial experimental or clinical evidence [78, 109]. Together these examples demonstrate how tumor type, microenvironmental context, and immune cell crosstalk determine the functional outcomes of mast cell and basophil activity.

### 5.1 Protective Functions of Basophils, and Mast Cell‐Driven Angiogenesis, in Skin Cancer

*Basophils:* Basophils have been implicated in immune‐protective mechanisms against skin carcinogenesis following DNA damage and epithelial barrier disruption. Specifically, basophil infiltration accompanied by local B cell class‐switching to IgE has shown protective effects against epithelial carcinogenesis [110]. IL‐33 can activate basophils; in experimental systems, IL‐33 stimulation induces CD63 upregulation and granzyme B, consistent with the induction of anti‐tumor mechanisms in the TME [111, 112].

*Mast cells:* Mast cells in the TME can induce complement activation which could exert critical anti‐tumor functions by inducing tumor cell cytotoxicity through complement‐dependent cytotoxicity (CDC) [42, 43]. The expression of complement component C3 in melanoma, however, is reported to promote tumor growth, angiogenesis, metastasis, immunosuppression, and inhibition of T cell responses has begun to be explored [113]. In melanoma mouse models, complement component C3 expression also correlated with poorer prognosis and treatment resistance to anti‐programmed death ligand 1 (PD‐L1) therapy [114].

Mast cells have also been implicated in human melanoma progression via alternative activation pathways, such as IL‐33/ST2 receptor signaling [40]. Mast cell infiltrates situated at melanoma tumor margins promote angiogenesis [69, 111], pointing to micro‐localization as potentially a key determinant of function and contribution to tumor progression and metastatic potential [115]. Human melanoma–associated mast cells display a distinct transcriptional signature marked by downregulation of FcεRI signaling, indicating a shift away from classical IgE‐mediated activation. Alongside this, melanoma‐associated mast cells feature altered expression of proteases and pro‐angiogenic mediators and upregulation of complement component C3, which becomes especially enriched in stage IV disease. This C3‐high mast‐cell phenotype correlates tightly with the mast‐cell marker TPSAB1 and is associated with significantly poorer patient survival. These reflect TME conditioning by melanoma‐secreted factors such as TGF‐β, IL‐33, and IL‐1β [69]. In a humanized melanoma xenograft mode, using immune‐deficient NOD‐*scid* IL2Rγ^null^ (NSG) mice, tumor‐infiltrating mast cells clustered in specific micro‐regions that were unresponsive to anti‐PD‐1 therapy, where they co‐localized with FOXP3^+^ Tregs and reduced CD8^+^/granzyme B^+^ T cell infiltration [41]. Mast cell‐rich regions also showed reduced HLA class I expression compared with regions that lacked mast cells. Combining anti‐PD‐1 treatment with c‐Kit inhibitors sunitinib or imatinib, which deplete mast cells by blocking c‐Kit–dependent survival, restored CD8^+^ T cell activity and enabled complete tumor regression [41]. These findings identify mast cells as key mediators of immune‐checkpoint resistance [34]. Emerging evidence therefore indicates basophil interactions with T and B cells leading to pro‐inflammatory effects and adaptive immunity, while mast cells may contribute to tumor progression and immunotherapy resistance in skin malignancies such as melanoma.

### 5.2 Mast Cells and Basophils as Immune Modulators and Markers of Prognosis in Pancreatic Ductal Adenocarcinoma (PDAC)

*Basophils:* In pancreatic ductal adenocarcinoma (PDAC), the proportion of basophils in tumor‐draining lymph nodes (TDLNs) was reported as an independent negative prognostic factor post‐surgery [49]. Mechanistically, basophils are recruited to TDLNs by chemokines such as CCL7/MCP‐3, secreted by alternatively activated monocytes, and once in the TDLN the basophils are activated by T cell‐derived IL‐3, leading to the subsequent release of IL‐4. This cascade contributes to an overall Th2‐driven immune microenvironment. It has also been shown that within mouse models, basophil depletion resulted in partial resistance to tumor engraftment, pointing to pro‐tumor roles [49].

*Mast cells:* Mast cells are reported to infiltrate the PDAC tumor stroma, correlating with tumor progression, metastasis, and angiogenesis. They facilitate a tumor‐supportive microenvironment by synthesizing and releasing pro‐angiogenic factors such as VEGF, FGF‐2, and tryptase. Therapeutic or genetic depletion of mast cells in experimental PDAC models demonstrated reduced tumor growth and enhanced survival outcomes [47, 48].

Together, in relation to PDAC, the presence and functions of basophils and mast cells correlate with tumor progression and outcomes, likely reflecting a Th2‐skewed environment.

### 5.3 Multifaceted Mast Cell Contributions and Tumor‐Promoting Roles of Basophils in Lung Cancer

*Basophils:* Basophils have been detected within lung tumor lesions and reported to have acquired distinct cytokine expression profiles likely influenced by local microenvironment cues. Cytometry‐by‐time‐of‐flight (CyTOF) studies on early lung adenocarcinoma samples identified basophils in both tumor lesions and non‐involved lung tissues of patients, although basophils were less frequent in tumor lesions compared to normal tissues and circulation [66]. Moreover, galectin‐3 secreted by lung adenocarcinoma cells has been shown to activate basophils in an antigen‐independent manner, enhancing their production of Th2 cytokines, IL‐4 and IL‐13, which in turn may contribute to tumor‐associated inflammation and immune modulation [54, 66, 116, 117]. These findings suggest a functional crosstalk between basophils, immune cells, and tumor cells that may contribute to Th2‐polarized inflammatory processes observed in lung adenocarcinoma. In contrast to basophils, mast cells in lung cancer display distinct phenotypic heterogeneity and have been more extensively characterized at both molecular and clinical levels.

*Mast cells:* Mast cell molecular subtypes are associated with clinical outcomes in early‐stage lung cancer, with mast cell abundance and molecular signatures also correlating with prolonged survival in early‐stage lung adenocarcinoma (LUAD) patients [118]. Mast cells in lung cancer are enriched at tumor margins and perivascular regions, where their mediator release contributes to angiogenic and pro‐coagulant remodeling of the TME [50].

In support of this notion, a study showed that lung cancer–derived exosomes are taken up by mast cells, triggering their activation and altering their cytokine secretion profile toward pro‐coagulant and thrombosis‐associated mediators. These exosome‐activated mast cells released factors linked to thrombosis and neutrophil extracellular trap–associated gene signatures [50]. In a different study, exosomes derived from lung cancer patients were found to contain SCF which can activate mast cells. Lung cancer‐derived exosomes can activate mast cells via SCF–Kit signaling, inducing tryptase release that reinforces pro‐angiogenic and pro‐metastatic remodeling within the TME [51]. Combined, these findings suggest that cancer exosome‐conditioned mast cells contribute to the cancer‐associated coagulation disorders common in lung cancer patients.

Phenotypic analyses revealed tumor‐associated mast cell (TAMCs) subsets in non‐small cell lung cancer (NSCLC) with a distinct phenotype in comparison to mast cells found in non‐malignant parts of the lung and identified two TAMCs subpopulations based on CD103 expression (CD103^+^ or CD103^‐^) [52]. Compared with CD103^‐^ counterparts, mature CD103^+^ TAMCs interacted with CD4^+^ T cells to a greater extent and were localized closely with cancer cells. Despite no prognostic advantages being correlated with a high frequency of CD103^+^ TAMCs, a high frequency of total TAMCs correlated with superior OS and PFS. This points to the presence, and potentially differential functions of TAMCs subpopulations in some lung tumor types and underlines mast cell heterogeneity [52].

Collectively, basophils in lung cancer appear to contribute predominantly through Th2‐associated cytokine production and tumor‐associated inflammation, whereas mast cells demonstrate broader functional heterogeneity, including roles in vascular remodeling, coagulation, and immune regulation.

### 5.4 Basophils and Mast Cells in Gastric Cancer

*Basophils:* Mechanistic data on basophils effector functions in gastric cancer remains limited compared to mast cells, with current evidence primarily associative. Peripheral blood basophil counts, as seen within gastric cancer, positively correlated with an increased accumulation of tumor infiltrating basophils, which were often observed in close spatial proximity to M2‐like macrophages [58]. These implicate a coordinated role between these cells in the TME of gastric cancer, consistent with similar observations in lung cancer (Table 1). Basophils in gastric cancer have primarily been described as immunomodulatory correlates, rather than as direct effectors of tumor angiogenesis.

*Mast cells:* By comparison, mast cells in gastric cancer have been more directly implicated in tumor vascularization and immune checkpoint modulation. Mast cells have also been reported in gastric cancer progression operating through diverse mechanisms. They aggregate around blood vessels and glands, where they promote tumor vascularization [119], consistent with their established pro‐angiogenic mediator profile [120, 121, 122]. Mast cells accumulate in the stroma surrounding tumors, where they secrete angiogenic cytokines (TGF‐β, TNF‐α, IL‐8, VEGF, FGF‐2) and the serine proteases tryptase and chymase, which are also implicated in both normal and tumor‐associated neo‐angiogenesis [123].

Mast cell density has been correlated with increased angiogenesis and tumor progression within patients with gastric carcinoma [119, 123]. Additionally, tryptase‐positive mast cells and c‐Kit receptor expressing cells also correlated with increased angiogenesis and lymph node metastasis in gastric cancer [119]. In concordance, a positive correlation was reported between tryptase positive mast cells in gastrointestinal tumors and the number of metastatic lymph nodes [123]. In specimens from 102 patients with gastric cancer, it was found that the average number of mast cells and blood vessels in the patient samples were significantly higher than those found in normal gastric tissue [124]. Together, these findings support the idea that mast cells contribute to blood vessel formation, tumor vasculature, linked with tumor progression, in gastric cancer, with mast cell localization near the newer vessels around gastric cancer cells.

Mast cells are also reported to activate pathways involving TNF‐⍺ and PD‐L1 that in the gastric cancer context can lead to suppressed immune responses and enhanced tumor progression. Tumor‐derived adrenomedullin was also reported to induce mast cell degranulation and promote tumor growth [125, 126]. Accordingly, mast cells could serve as potential targets alongside immunotherapy: in a human model of gastric cancer, inhibition of mast cell‐associated PD‐L1 resulted in reduced tumor growth with an increase in T cell activation [34].

Conversely, mast cell‐derived tryptase can serve as a positive prognostic marker in gastric cancer [57, 127]. In one clinical study, tryptase density was assessed through immunohistochemistry and the correlation between tryptase expression and prognosis was evaluated. Stage I patients who had higher levels of tryptase expression in tumor tissues had superior OS and PFS [57].

While basophils in gastric cancer appear to correlate with a Th2‐polarized immune microenvironment, mast cells have been more directly associated with angiogenesis, protease‐mediated matrix remodeling, and modulation of immune checkpoint pathways such as PD‐L1.

### 5.5 Basophils and Mast Cells in Colorectal Cancer Progression, Angiogenesis and Immune Modulation

*Basophils:* Circulating basophil levels have been associated with colorectal cancer prognosis (Table 1). Lower basophil blood counts correlate with advanced tumor stages (T stage) and higher lymph node involvement (N stage), increased venous and perineural tumor invasion, and poorer OS [128].

*Mast cells:* The prognostic significance of mast cells in colorectal cancer remains controversial, with some studies reporting correlations with improved prognosis, whereas others report no significant associations [129, 130, 131]. In a model of colorectal cancer, highly suppressive Tregs were shown to be unable to suppress degranulation of human LAD2 mast cells [132]. This suggests a complex interaction between mast cells and Tregs which may influence tumor immune evasion mechanisms [45].

## 6 Concluding Remarks and Future Directions

Basophils and mast cells, long regarded primarily as mediators of hypersensitivity and IgE‐mediated allergic responses, are now recognized as immune regulators with diverse and sometimes opposing roles in cancer. Their influence within the TME extends across angiogenesis, immune modulation, ECM remodeling, and direct cytotoxicity, with their ultimate impact determined by factors such as tumor type, stage, and the local immune contexture.

Emerging literature demonstrates that these cells can either foster tumor progression, through pro‐angiogenic signaling, immunosuppression, and promotion of Th2‐skewed inflammation, or contribution to tumor control, via recruitment and activation of effector immune cells, degranulation, and modulation of adaptive immunity. The interplay of these functions may have tangible consequences for patient prognosis and therapeutic response. For instance, basophil infiltration in the lymph nodes of patients with pancreatic cancer correlates with poorer outcomes, while mast cell density in certain cancers, such as melanoma, has been correlated with resistance to immunotherapy. Such heterogeneity presents both a challenge and an opportunity. Understanding the precise circumstances in which basophils and mast cells act as tumor‐promoting or tumor‐inhibiting agents will be critical to developing targeted interventions. Deep multiparameter profiling of these cells, incorporating detailed functional evaluations through assays such as the BAT and MAT, are important for therapy design and selecting appropriate combination therapy.

Overall, emerging evidence has positioned basophils and mast cells as critical, yet underexplored, players in cancer, with multifaceted roles spanning angiogenesis, inflammation, and therapy resistance. Whilst foundational knowledge of basophil and mast cell interactions emerge arising from their established roles in mediating both allergic and hypersensitivity reactions, significant gaps remain in our understanding of their phenotypes, activation states, interactions, and complex functions in cancer progression and metastasis. The future of AllergoOncology lies in addressing these gaps and leveraging the effector and allergic mechanisms of these cells to target cancer, through innovative clinical applications to better inform treatment outcomes and patient prognosis. The application of BAT and MAT offer the possibility to help predict hypersensitivity reactions, guide immunotherapy selection, and refine prognostic assessment of current and experimental therapeutics. The BAT and MAT could be established as standardized procedures within clinical practice for predicting and monitoring immunotherapy‐induced hypersensitivity, considering the emergence of IgE‐based therapeutics in clinical studies. However, this will require overcoming current barriers, including technical difficulties in cell isolation and manipulation, as well as lack of sufficiently robust assay standardization for clinical use.

Basophils and mast cells are emerging as clinically relevant regulators at the interface of allergy and cancer. Standardized functional profiling using BAT and MAT offers a pragmatic route to translate mechanistic insights into clinical utility by predicting hypersensitivity risk, informing immunotherapy selection, and refining prognostic assessment. Integrating these assays into clinical practice could support more precise and biologically informed cancer care, positioning AllergoOncology as a tangible framework for next‐generation immunotherapy strategies.

## Author Contributions

**Jack Alder:** methodology, writing – original draft, writing – review and editing, investigation. **Katie E. Lacy:** writing – review and editing. **Debra H. Josephs:** writing – review and editing. **James Spicer:** writing – review and editing. **Heather J. Bax**: writing – original draft, writing – review and editing. **Sophia N. Karagiannis:** conceptualization, methodology, investigation, writing – original draft, writing – review and editing, project administration, funding acquisition. **Jitesh Chauhan:** conceptualization, methodology, investigation, writing – original draft, writing – review and editing, project administration.

## Funding

The authors acknowledge support by the Wellbeing of Women (Grant RG2562); the British Skin Foundation (Grant 006/R/22); Worldwide Cancer Research (Grant 24‐0087); Breast Cancer Now (Grant KCL‐Q4‐Y1); the Guy's and St Thomas's Foundation Trust Charity Melanoma Special Fund (Grant 573). This research was supported by the King's Health Partners Centre for Translational Medicine. The views expressed are those of the authors and not necessarily those of King's Health Partners.

## Conflicts of Interest

S.N.K. and J.S. are founders and shareholders of Epsilogen Ltd. S.N.K., J.S., D.H.J., and H.J.B. declare patents on antibodies for cancer. J.C. and H.J.B. have been employed through a fund provided by Epsilogen Ltd. All other authors declare no conflicts of interest.

## Data Availability

Data sharing not applicable to this article as no datasets were generated or analyzed during the current study.
